# How TGfU Influence on Students’ Motivational Outcomes in Physical Education? A Study in Elementary School Context

**DOI:** 10.3390/ijerph18105407

**Published:** 2021-05-19

**Authors:** Vicente Gaspar, Alexander Gil-Arias, Fernando Del Villar, Alba Práxedes, Alberto Moreno

**Affiliations:** 1 Faculty of Physical Activity and Sports Sciences, University of Extremadura, 10003 Cáceres, Spain; vicentegaspargil@gmail.com (V.G.); amorenod@unex.es (A.M.); 2 Centre for Sport Studies, Rey Juan Carlos University, Fuenlabrada, 28942 Madrid, Spain; fernando.delvillar@urjc.es; 3 Faculty of Life Sciences and Nature, University of Nebrija, 28248 Madrid, Spain; apraxedes@nebrija.es

**Keywords:** Self-Determination Theory, basics psychological needs, questioning, gender

## Abstract

The purpose of this study was to implement a comprehensive teaching program based on the principles of Teaching Games for Understanding (TGfU) model and questioning, and to assess its consequences for students’ satisfaction of basic psychological needs, motivation, perceptions of ability and intention to be physically active during Physical Education lessons in primary education. A quasi-experimental design was utilized. Participants were 111 students from two different groups of fifth and sixth graders, all enrolled in one primary school. Participants were divided into experimental and control group. Experimental group experienced a TGfU unit, according to small side games and the questioning. Control group experienced a small side games unit, without questioning. Within-group results showed that experimental group students reported significantly higher mean scores in all dependents variables of the study, in both genders. Results showed that control group only reported significantly higher mean scores in intention to be physically active variable, also in both genders. The results demonstrate the need to implement didactic units under comprehensive pedagogical approaches to improve motivation and the intention to develop healthy lifestyle habits in female and male students. More researches are needed to support this evidence.

## 1. Introduction

Currently, the school must be a context that favors the development of the key competencies and autonomy of the students, in order to guide them towards choosing appropriate behaviors for life. In this sense, Physical Education (PE) becomes a fundamental area to promote active behaviors in students by creating healthy lifestyle habits and active occupation of free time stand out.

Therefore, teachers play a key role in the teaching/learning process because they project, with their way of being and the way they work, all the influences that students receive during lessons [1]. In PE, and specifically in elementary PE, there have been recent developments about the type of work required from PE teachers [2]. Indeed, some works suggest that PE teachers should be trained to personalize their styles of intervention and to use them to improve students’ motivation [3]. This improvement can help achieve appropriate active behaviors.

In scientific literature, one of the theories that have analyzed motivation and its consequences in an educational context is the Self-Determination Theory (SDT) [4,5]. This theory states that people strive to meet their basic psychological needs (BPNs) for autonomy (humans’ need to experience a sense of willingness in their actions), competence (humans’ need to develop a feeling of mastery through interacting with the environment) and relatedness (humans’ need to interact with the other individuals) [6]. Central to the theoretical framework of SDT is the distinction between different types of motivation that vary according to one’s level of self-determination. SDT proposes that motivation lies along a continuum in which three levels of self-determination are distinguished: autonomous motivation; controlled motivation; and amotivation [4]. When students show more autonomous levels of self-determination, they present greater possibilities of adaptation and enjoyment in PE practice. Conversely, if students experience less autonomous levels of self-determination, there is less chance that students will enjoy practicing and, consequently, demonstrate greater demotivation and boredom in PE practice [7].

Adequate BPNs satisfaction is essential for optimal functioning, adaptive social development and personal wellbeing [8]. Studies on SDT have shown that PE teachers are responsible for generating positive experiences among students [9]. This fact is linked to a greater sense of enjoyment during PE lessons, the inclusion of regular physical exercise practice in daily routines, and the appreciation of physical activity for its associated benefits [10].

However, teachers should aim for students to participate in their classes with an autonomous motivation; that is, teachers should aim for students to make voluntary contributions due to the interest, satisfaction or pleasure they experience when engaged in class activities. This type of motivation is preferable because it is linked to outcomes such as effort, concentration, vitality, positive development, and the intention to be physically active [11]. Thus, enhancing this type of motivation influences personality and can help individuals attain lasting motor practice [12].

With this considered, the Organic Law of Education 2/2006, Article 1, states that the Spanish educational system is inspired by several principles, among them an emphasis on individual effort and student motivation. Motivated students tend to try hard on tasks, which favor better sports practice, and have more interest in doing things well, meaning they can improve the skills they learn. This highlights the relationship between motivation, practice of physical activity, effort, skill, competence and efficiency [13]. Students who demonstrate ability, competence and self-efficacy can give themselves positive feedback, elevate their motivation and, consequently, their interest and effort in what they do [14].

Accordingly, current guidelines are that teachers should promote in their students a perception of ability in PE, because this perception of ability encourage students’ predisposition to participate in any sport outside the school context. In this regard, several studies show that an intention to be physically active in the future is predictive of physical activity practice [15,16]. It is, therefore, very important that the benefits of practicing physical activity are instilled from an early age [17,18]. PE teachers are good promoters of the intention to practice physical activities, to keep students active [19] and, therefore, for increasing the practice of regular physical activity. Moreover, past research shows that the intention to be physically active is closely related to high levels of autonomy, competence and relatedness [20], and to more self-determined levels of motivation [21,22,23,24].

As such, we emphasize that elementary PE allows students to experience positive experiences in an educational environment, thus helping to improve their interpersonal relationships and promoting the acquisition of responsibilities and problem-solving. However, these positive experiences are sometimes different depending on gender. Several studies have found that the female gender presents lower levels of motivation than the male gender in the practice of physical activity in PE classes [25]. Therefore, we consider essential the opinion of the female on PE contents to satisfy their BPNs. The teaching programs must respond to less stereotyped content, favoring a more satisfactory practice of physical activity, for girls as well [26]. For this reason, we consider it essential that the research should be oriented towards establishing a comparison based on the gender of the different psychological variables that influence the teaching–learning process.

The teaching–learning process must be student-centered, so the teacher should use pedagogical models that favor this fact. PE teachers have to design student-centered learning settings based on students’ needs, that allow psychomotor, cognitive, and affective development [27]. In this regard, Teaching Games for Understanding (TGfU) pedagogical model considers the students’ needs, while also providing a learning environment that prioritizes student motivation, problem-solving and decision-making [27]. This model allows students to assimilate the tactical aspects of the sport by playing the game in small-sided and/or modified/conditioned [28]. Problem solving in a changing game environment is critical to the TGfU pedagogical model [29], and therefore one of its objectives is to orient students toward the analysis of different game situations. For this, the application of the model must follow several phases [30,31]: (1) students must first be able to understand the game form, introducing them to a variety of game forms in accordance with their age and experience; (2) later, the students should learn to appreciate the game, understanding the roles of the game that must be played; (3) once they understand the rules, it is important that students acquire a tactical awareness. In this phase, the decision-making process developed by the students allows, both them and the teacher, to recognize and attribute tactical deficiencies; (4) finally, in the context of the game, the students must execute specific technical skills of the sport practiced.

In this way, the TGfU model encourages the simultaneous development of physical, cognitive and emotional skills and to promote social, physical and cognitive learning alongside tactics in contextualized situations using the pedagogical principles of sampling, modification (representation and exaggeration) and tactical complexity [29,32]. These pedagogical principles must be taken into account in the design of the learning tasks [33]: (1) sampling, which is achieved through the use of the global game, finding the tactical aspects common to the different sports; (2) representation, consisting of the adaptation of the games to students’ developmental needs, keeping the tactical structure; (3) exaggeration, raising possibility of including new rules or modifying them to help assimilate tactical contents; (4) tactical complexity, posing the learning tasks in progression of tactical difficulty.

It is not only important to consider these pedagogical principles and respect the development phases of the TGfU model. In addition, the questioning is a fundamental tool within TGfU aimed at improving student’s ability to reflect on their own sports practice [34,35]. In this context, teachers serve as guides that help students solve tactical problems that occur during the game. Over time, teachers progressively reduce their help so that students gain autonomy and accountability [36,37]. Teachers should ask questions during the game, while letting students continue to play, and then resort to small debates that stimulate tactical thinking by helping students to analyze the game and to seek solutions in practice [28,38]. Teachers should encourage the exchange of questions and answers because it encourages discussion about sports games [39].

At this point, how should the teachers implement this questioning? The questions used by the teachers need to stimulate thinking and social interaction from which learning emerges [40]. In this sense, the literature establishes different methods to implement questioning in classes, without the need for the teacher to have an excellent knowledge of the content or a perfect capacity for observation and analysis [41]. Therefore, the GROW model highlights the need to introduce four steps in questioning [42]. The first step is to establish the goal (G) of the activity, and then to implement the questioning at three different levels: examine the reality (R, describe their current reality); explore the options (O—discuss what to do and how to do to achieve the objective of the proposed task); and establish the will/way forward (W—discussion is converted into a decision/action plan for the next bout of game play).

As previously commented, the questioning is a powerful tool for the development of independent and emergent decision making in sports [36]. Therefore, by structuring opportunities for questioning episodes within PE lessons, the teacher can guide, facilitate and scaffold the learner’s problem solving capability [43]. Improving this ability will allow students to be more autonomous in their future decisions. In addition, the interactions generated between the students and between the students ant the teachers, will help develop empathy (and relatedness) with each other [44].

Despite the academic popularity of the TGfU model, its inclusion in some official curricula does not appear to match with to an equivalent inclusion in school PE for most countries in which it has been disseminated [45,46]. In other words, the use of the TGfU model in teaching practice, and particularly at the elementary education stage, is still very limited [47]. As such, the present study is important because its results may help improve the teaching–learning process in an elementary PE context. Specifically, the implementation of intervention programs based on the TGfU model and questioning will help increase student motivation via more game involvement, increase student enjoyment of game practice, improve competence satisfaction, and thus elevate student’s intentions to by physically active in PE classes [48]. Moreover, PE teachers’ use of the TGfU model and questioning will allow design an inclusive learning environment where all students (boys and girls) could increase their motivational processes and enjoyment within PE lessons. In this regard, the purpose of the current study was to implement an intervention based on pedagogical principles of TGfU and questioning, and to assess its consequences on students’ BPNs satisfaction, motivation, perceptions of ability and intention to be physically active during PE lessons in elementary education.

## 2. Materials and Methods

### 2.1. Design and Participants

The study was conducted in an intact educational context and a convenience sampling was used. Participants were 111 elementary school students (*M_age_* = 10.95, *SD_age_* = 0.64) from two different groups of fifth and sixth year of elementary school in south-west Spain and members of four already established classes. Fifty-four students were taught through a TGfU unit combined with questioning (experimental group), while 57 students only received a TGfU unit without the application of the questioning (control group) within a pre-test/post-test quasi-experimental design. In each year of elementary school (fifth and sixth), classes were randomly assigned to experimental group and control group. Thus, each group was made up of students from fifth and sixth year of elementary school. None of the students had previously received a TGfU model-based teaching unit in their PE lessons. The interventions were conducted by the same teacher, who was male and had 15 years of elementary school teaching experience.

The research has been developed under the ethical guidelines of the Declaration of Helsinki. The participants and their parents were informed of the study. As the participants were underage, the parents signed an informed consent if they agreed to participate in the study. The research project was fully approved by the ethics research committee of a Spanish university.

### 2.2. Instruments

#### 2.2.1. Basic Psychological Needs Satisfaction

The Spanish version for PE context [49] of the Basic Psychological Needs in Exercise Scale (BPNES) was used to measure the satisfaction of the BPNs. The scale begins with the initial statement “In my PE lessons …”. This instrument contains 12 items grouped into three factors (four items per factor) that measure autonomy satisfaction (e.g., “We carry out exercises that are of interest to me”), competence satisfaction (e.g., “I carry out the exercises effectively”), and relatedness satisfaction (e.g., “My relationship with my classmates is friendly”). Cronbach’s alpha values were adequate (pre/post autonomy satisfaction: 0.73/0.67; pre/post competence satisfaction: 0.73/0.70; pre/post relatedness satisfaction: 0.79/0.75).

#### 2.2.2. Motivation

The Motivation in Physical Education Questionnaire in Primary Education (CMEF-EP) was used [50]. This instrument contains 18 items (10 items per autonomous motivation, 4 items per controlled motivation and 4 items per amotivation) that follow the initial statement “I take part in the PE lessons …” and measure: autonomous motivation calculated through the mean score of intrinsic regulation (4 items) (e.g., “because I enjoy learning new skills”), integrated regulation (4 items) (e.g., “because I believe that physical education is according with my values”), and identified regulation (2 items) (e.g., “because I feel bad if I don’t participate in the activities”) [43]; controlled motivation was calculated through the score of external regulation items (e.g., “because I want the teacher to think that I am a good student”) [51]; amotivation was calculated through the score of amotivation items (e.g., “I don’t know clearly because I don’t like anything”). Cronbach’s alpha values were adequate (pre/post autonomous motivation: 0.80/0.77; pre/post controlled motivation: 0.74/0.77; pre/post amotivation: 0.71/0.69).

#### 2.2.3. Perception of Ability

The Spanish version for PE context [13] of the Physical Education Predisposition Scale (PEPS) [52] was used. This instrument contains 11 items that follow the initial statement “In relation to PE lessons…” and measure the perception of effort (6 items) and perception of ability (5 items). In this study, only perception of ability was measured (e.g., “I’m very capable at PE”). Cronbach’s alpha values were adequate (pre/post: 0.71/0.72).

#### 2.2.4. Intentions to Be Physically Active

The intention to be physically active scale for elementary education context [15] was administered to participants. The instrument contains 5 items and measure a single factor (e.g., “In addition of PE lessons, I like to practice sports”). Cronbach’s alpha values were adequate (pre/post: 0.71/0.70).

### 2.3. Procedure

Prior to the study, it was necessary to conduct a period of training with the PE teacher that lasted three sessions, each lasting 90 min. The first session addressed the pedagogical principles of the TGfU model (modification representation, modification exaggeration, and tactical complexity), the second was related to the application of small-sided games, and the third was related to the use of questioning in student training [53]. These training sessions were led by the first author, who has extensive experience and knowledge in TGfU pedagogical model in elementary PE. Before starting the intervention, the PE teacher carried out two PE lessons with two different classes of students that did not participate in this study. After each teaching session, both of which were observed by the first author, a post-lesson reflection meeting was held to discuss strengths and areas in which both the teacher and first author felt the sessions could be improved. During these reflection meetings, the first author linked discussions to the TGfU model benchmarks seen in Table 1. In these lessons, the researcher was in a discrete location and did not intervene in practice.

When the teacher training process was completed a data collection was conducted with all the students participating in the study (pre-test). This occurred in the week prior to the starting of the intervention. Students were required to answer the questionnaires provided by the researcher independently, without additional help to that provided on the instrument itself. All students completed the instruments in a 15–20-min period in the absence of the PE teacher. After pre-test, students were exposed to the 16 learning sessions of the intervention program. All students experienced the same learning activities, although those in the control group did not have the application of questioning. The groups for these sessions were determined by the teacher based on the development and evolution of the activities. After the intervention, the ultimate data collection phase (post-test) was conducted following the same procedure as pre-test.

### 2.4. Intervention

The intervention program was conducted in the second trimester, in accordance with the timing provided for this content in the center’s PE academic curriculum.

We used a didactic structure based on the TGfU pedagogical model to design the intervention program activities for the experimental group. Tactical variables that allowed the game to be modified and to develop in complexity were manipulated (game rules, number of students per team, level of opposition, court size, the baskets, the balls, and the game duration). Initially, the number of team components was minimal, the size of the court was not limited, and there was no time limit to achieve the objective. As such, the game allowed for the maximum participation of students and greater game continuity, had a lower tactical demand, and made it easier for students to perform skills.

Each scheduled session began with a five-minute modified game, which encouraged students to reflect on a specific problem defined by the teacher. Next, students engaged in three modified games of ten minutes each. These games had a greater tactical component focused on the basic principles of attack and defensive gameplay [54]. The games were modified to be representative. For example, we defined a common space for all groups, and they had to maintain control of the ball through passes and receptions while progressing towards the basket and avoiding having the ball stolen. The team that performed ten passes in a row without losing control of the ball were awarded a point. These modified games were complemented by the practice of games modified by exaggeration (e.g., 3 vs. 3 with only forward passes allowed). In this way, the sessions allowed students to experience a high number of specific game situations in a realistic context [55].

Within the context of modified games, questioning was intended to cognitively engage the students. For each modified game, the teacher questioned students on the technical-tactical principles being employed. To confirm the effectiveness of the questioning, we considered five areas that ensure the quality of the teacher’s intervention (strategy, tactics, technique, standards, psychological aspects) [56]. The form of the question (What? Where? When? Why? Who? How?) [57] was also considered. As an example, for a 2 vs. 1 scenario where the objective was move towards the opposite basket with a low level of opposition the question would be phrased as follows: What should you do to make a basket? Thus, the intention is to guide the student at three levels of questioning: time, space, and level of risk [58].

The sessions were organized according to the main content to be worked on. The first and second sessions were dedicated to working on ball possession in attack and the dribble. The third and fourth sessions were dedicated to the pass, and the fifth and sixth sessions dedicated to shooting. Sessions seven and eight were focused on the spaces. In the ninth and tenth sessions, students worked on defense, and in the 11th and 12th sessions, attack. Sessions 13 and 14 worked jointly on attack and defense, and sessions 15 and 16 worked on integrating shooting, passing, dribbling, displacements, attack and defense.

The sessions for the control group had the same content, sequencing, structure and games as the experimental group, but the teacher did not apply the intervention based on questioning.

### 2.5. Instructional and Treatment Validity

The fidelity of the interventions was assessed using a checklist (Table 1) [59]. All items enabled researchers to measure PE teacher fidelity to the characteristics of TGfU and questioning (experimental group), while checklist items 1, 2, and 3 helped researchers examine teacher fidelity to TGfU unit (control group). The fidelity assessment was based on direct and external systematic observation. The first author and one additional observer with experience in pedagogical models in PE observed a sample of six sessions for each pedagogical approach, more than 12.5% the total sample [60]. One hundred percent agreement was reached between the two observers. They reported on the facets of the pedagogical models that were present in the lessons before reporting on the outcomes of the intervention. Each observer therefore confirmed that all key aspects included in the instructional checklist were performed by the teacher in each of the observed lessons.

### 2.6. Data Analysis

The statistical program IBM SPSS v. 24.0 was used for data analysis. Preliminary assumption testing was conducted to check for normality, homogeneity of variances, and multicollinearity. Levene and Kolmogorov–Smirnov tests were performed to confirm the assumptions of homogeneity of variances and normality of distribution, respectively (*p* > 0.05). The assumption of multicollinearity was deemed to have been met, as no Spearman values for the dependent variables in both the pre-test and post-test measures were over 0.70. For each group and gender at each of the two different time points, mean and standard deviations were calculated. To compare between-groups and within-group differences, a 2 × 2 × 2 within-pedagogical approach (TGfU unit with questioning and TGfU unit without questioning) × test time (pre-test and post-test) × gender (boys and girls) MANOVA was conducted. A Bonferroni correction factor was used for these analyses to control for Type 1 errors due to using multivariate comparisons. If an overall multivariate effect was significant, the univariate ANOVAs were interpreted for both genders to examine which specific constructs contributed to the overall multivariate effect. Effect sizes were calculated using the partial eta-squared statistic (ηp^2^) which provided an insight into the magnitude of the differences found. Effect sizes above 0.01 were considered small, above 0.06 medium, and above 0.14 large [61]. The level of statistical significance was established at *p* ≤ 0.05 (95% confidence interval).

## 3. Results

### 3.1. Pre-Test Analysis

In pre-test, Levene tests were performed to confirm the assumptions of homogeneity of variances (*p* > 0.05). The results showed no significative differences in both group in all variables considered (autonomy, *p* = 0.925; competence, *p* = 0.233; relatedness, *p* = 0.904; autonomous motivation, *p* = 0.870; controlled motivation, *p* = 0.872; amotivation, *p* = 0.086; perceptions of ability, *p* = 0.471; intention to be physically active, *p* = 0.909).

### 3.2. Between-Group Post-Test Analysis

In post-test, a significant multivariate effect was not found for both boys (Wilks’ Lambda = 0.96; *F*_(8, 100)_ = 0.49; *p* = 0.856; *η*_p_^2^ = 0.03) and girls (Wilks’ Lambda = 0.89; *F*_(8, 100)_ = 10.54; *p* = 0.184; *η*_p_^2^ = 0.10).

### 3.3. Within-Group Pre-Post-Test Analysis

Within-group multivariate contrasts showed a significant effect with a higher effect size in boys (Wilks’ Lambda = 0.63; *F*_(8, 100)_ = 19.16; *p* < 0.001; *η*_p_^2^ = 0.36) than girls (Wilks’ Lambda = 0.70; *F*_(8, 100)_ = 5.26; *p* < 0.001; *η*_p_^2^ = 0.29), who were taught under the TGfU unit with questioning0. In the pairwise comparisons, both boys and girls reported significantly higher values on all the dependent variables in the post-test compared to the pre-test, except controlled motivation for girls and amotivation for both boys and girls (Table 2). Moreover, a significant multivariate effect was not found for both boys (Wilks’ Lambda = 0.87; *F*_(8, 100)_ = 1.75; *p* = 0.096; *η*_p_^2^ = 0.12) and girls (Wilks’ Lambda = 0.92; *F*_(8, 100)_ = 1.05, *p* = 0.398, *η*_p_^2^ = 0.07) taught under the TGfU unit without questioning.

## 4. Discussion

The aim of the present study was to implement an intervention based on pedagogical principles of TGfU and questioning, and to assess its consequences on students’ BPNs satisfaction, motivation, perceptions of ability and intention to be physically active during PE lessons in elementary education. Our results showed that boys and girls taught through the TGfU unit with questioning would report higher scores on all variables post-intervention compared to pre-intervention than boys and girls taught through TGfU unit without questioning. The TGfU unit without questioning group only showed significant differences in intention to be physically active variable after the implementation of the intervention program.

Regarding the autonomy dimension, the TGfU unit with questioning elicited greater student engagement in the different learning tasks. The fact that students exchanged ideas to solve the tactical problems posed by the teacher meant they were true protagonists of the teaching–learning process. These results are, therefore, consistent with past research that analyzed the effects of TGfU model on student autonomy in PE [62]. The principal aim of the TGfU model is to give students a leading role in their sports learning [63]. Thus, promoting student autonomy by using flexible teaching programs can help avoid student frustration [64,65] and the emergence of challenging behaviors [66,67].

Concerning the competence dimension, the application of the TGfU model meant that there were fewer teacher corrections; indeed, corrections were only given when students encountered major difficulties. In addition, it is shown that students receive more support from their peers when they make mistakes, as it is agreed with other students the degree of participation in the modified games conducted in the session [68]. Likewise, affective feedback (e.g., “Good!”, “Very good!”, “Come on!”) conveyed support for what the students were doing at that time of the game [69]. This positive reinforcement serves to increases students’ satisfaction of competence and thus increase their intrinsic motivation [67]. Equally, the questioning also allows students to discover their main strengths to solve different tactical situations and that through exploration and inquiry it is also possible to build greater tactical knowledge, and consequently make more adaptive decisions.

Regarding the relatedness dimension, the questioning used by the teacher as a pedagogical tool greatly facilitated the development of discussions between the students, thus promoting a greater amount of interaction. The TGfU model is one of the most widely used pedagogical models for PE interventions. This is because this model places the student as the main protagonist of the teaching–learning process via the assignment of responsibilities and by encouraging active participation during PE sessions [70].

Thus, by providing a climate of cooperation, motivation and sportsmanship [71], PE classes can be considered a great ally for promoting a positive setting that encourages the practice and assimilation of prosocial behaviors [72].

There are two primary explanations for the improvement in student autonomous motivation after the implementation of the program: (1) the program led to a progressive and adaptive assignment of decision-making and task-related responsibilities, and (2) the program caused an increased critical capacity in students via questioning. In this sense, the increase in students’ satisfaction of BPNs could promote elevated levels of self-determined motivation. Indeed, such a positive relationship has been demonstrated in numerous past studies [73,74]. It has been shown in several instances that adequate support for BPNs helps PE students develop more self-determined motivation [75,76]. As such, it is typically considered that the TGfU model is an ideal way to promote higher levels of motivation, which in turn help generate greater adherence to the practice of physical activity [77].

Concerning students’ perceptions of ability; the results of this study revealed significant differences between the groups, with the highest levels for the experimental group. This is likely because the intervention included a variety of tasks adapted to the students’ maturity and developmental levels, a progression in the teaching–learning process and the use of game forms that capture the essence of real games [78]. These conditions can enable learning based on important values such as effort and perseverance. Learning these values can generate a good predisposition towards learning new skills, thus encouraging students to achieve their goals [79]. As mentioned, a fundamental aim of PE should be to help students to be more motivated because the most motivated individuals feel more skilled, and this helps them to achieve better results in the teaching–learning process [80]. Therefore, increased participation and involvement in game forms helps support students’ perceptions of ability and encourages greater motivation for sports practice [81].

The study also found that students’ intentions to be physically active were favored by the implementation of the intervention program. To allow for the satisfaction of autonomy and accountability, students must be at the center of the teaching–learning process and understand that the practice of physical activity is a fundamental component of daily life [82]. In this sense, the application of the TGfU model will encourage students to have more positive attitudes toward continuing motor practice in the future [83]. Some past works have highlighted the relation between one’s intention to be physically active and the satisfaction of the BPNs [84,85,86]. Moreover, higher autonomous motivation is related to both more physical activity and more intention to engage in physical exercise in the future [21,23,24].

Regarding gender, the application to TGfU unit with questioning allows for the creation of varied learning situations in which boys and girls experience the same opportunities, and where all students are required to collaborate and share resources to optimize learning for both the self and others. Therefore, designing learning tasks that are linked to the reality of sport using the pedagogical principles of TGfU and questioning cause students to have a more positive image of sports practice [87]. The fact that this model promotes interpersonal relationships, interactions between team members and feelings of affiliation, union and friendship makes them an important pedagogical resource for meeting motor, social and affective goals between people of different genders and skill levels [32]. From the study results, it was evident that this program was highly inclusive given that the rules and materials were adapted to motivate girls and less skilled students [63], encouraging reflection during the practice of all students. Therefore, we will be avoiding the repetitive and boring sessions that drove students away from sports games, especially girls and less skilled individuals [88]. Several studies, based on the application of participative pedagogical models, have analyzed the effects produced on different motivational and psychological variables. Gil-Arias et al. [89] applied a PE training program, according to the pedagogical principles of TGfU. Although the results showed effective in both genders, a large effect size was found for girls. In the same line, another study analyzed the impact of a basketball unit taught using eigther, a hybrid TGfU/SE, or direct instruction model on perceived autonomy support, perceived NPBs and autonomous motivation [90]. Equally, the results showed that boys and girls who participated in the hybrid unit reported higher levels of autonomy support and autonomous motivation compared to boys and girls who participated in the direct instruction unit.

In sum, the TGfU unit with questioning has the advantage of allowing for a more inclusive practice environment by making the teaching process more interesting, understanding and fun for both male and female students [91].

Despite the findings described, some limitations and future research directions should be considered. First, the study sample was small. The study was developed in real context, with only one teacher. It is necessary in the future to train more teachers to be able to intervene in a larger sample. Second, the research did not consider a control group with an application based on traditional methodology. An investigation with three different treatment groups would allow to establish stronger conclusions. Third, the effects of only one unit (with questioning and without questioning) was examined in this study. Consequently, it would be valuable to replicate the current study and investigate the effect on psychosocial variables over a more longitudinal time frame with the application of consecutive TGfU units in different sports. Finally, to include data generated from qualitative methods (e.g., interviews) would allow to obtain more in-depth insights into students’ motivational processes in PE.

In this regard, future studies are necessary to acquire a deeper knowledge of this topic.

## 5. Conclusions

Several conclusions can be drawn, according to the results obtained in this study.

First, we consider that comprehensive questioning-based teaching programs are a teaching resources of enormous relevance in PE because: (a) they consider students as the central axis of the teaching–learning process, (b) they encourage autonomy as students participate in collective tasks and debates, and (c) technical and tactical demands are adapted to student characteristics. This allows students greater control of their behavior, a greater sense of group membership, and ultimately greater satisfaction in PE sessions. Therefore, the teachers can promote a more significant learning in your students.

As a second conclusion, we consider that this type of training program helps students to satisfy their BPNs, and consequently increases self-determined motivation. Equally, this type of program help students to have higher perceptions of ability and leads to increased satisfaction in PE classes, thus promoting more intention to practice physical activity in the future. Therefore, in this sense, we can conclude that teachers need to reconsider the planning their teachings. It´s essential to include alternative teaching methodologies with questioning to get more active and healthy students.

The last one and the most important conclusion of the present study is that comprehensive questioning-based teaching programs allow the possibility to create an inclusive context in PE where students have elevated perceptions of ability, gain more confidence, and achieve greater engagement in learning. Despite the existence of social and cultural stereotypes in terms of physical activity, if the teachers promote reflection and small side games in PE lessons, it will help all students have opportunities to increase their participation, social interactions and physical activity in the school and extralective context, regardless of their gender.

## Figures and Tables

**Table 1:** Instructional Checklist.

Date:	Present	Absent
1. All the tasks are related to the small-sided game that is being taught.		
2. Modifications to the full-game were performed.		
3. Students employed at least 30 min in the practice of modified games.		
4. Teacher used open-ended questioning to guide the students toward correct answers to the tactical problem.		
5. Teacher used individual and collective questioning according students’ needs.		
6. The questioning was applied at least two times during the practice.		

**Table 2:** Descriptive statistics and within-group analysis of each dependent variable.

Variables	Gender	Pre-Test TGfU Unit with Questioning	Post-Test TGfU Unit with Questioning	*p*	95% CI	Pre-Test TGfU Unit without Questioning	Post-Test TGfU Unit without Questioning	*p*	95% CI
		*M (SD)*	*M (SD)*			*M (SD)*	*M (SD)*		
Autonomy	Boys	3.17 (0.75)	4.15 (0.86)	<0.001	[−1.26, −0.688]	4.03 (0.68)	4.04 (.59)	0.936	[−3.18, 0.293]
	Girls	3.58 (0.76)	4.20 (0.60)	<0.001	[−935, −0.324]	3.59 (0.66)	3.71 (.61)	0.425	[−0.429, 0.182]
Competence	Boys	3.60 (0.50)	4.31 (0.73)	<0.001	[−969, −0.464]	4.30 (0.38)	4.15 (.39)	0.272	[−0.118, 0.414]
	Girls	3.55 (0.68)	4.35 (0.58)	<0.001	[−1.06, −0.530]	3.87 (0.59)	3.95 (.59)	0.536	[−0.349, 0.183]
Relatedness	Boys	3.84 (0.67)	4.30 (0.82)	0.002	[−749, −0.167]	4.30 (0.62)	4.22 (.68)	0.591	[−0.224, 0.390]
	Girls	3.89 (0.78)	4.52 (0.53)	<0.001	[−936, −0.323]	3.98 (0.79)	4.17 (.62)	0.212	[−0.501, 0.112]
Autonomous motivation	Boys	3.67 (0.64)	4.41 (0.63)	<0.001	[−1.03, −0.452]	4.25 (0.53)	4.34 (.48)	0.530	[−0.403, 0.209]
	Girls	3.77 (0.65)	4.40 (0.54)	<0.001	[−935, −0.324]	4.00 (0.67)	4.01 (.84)	0.952	[−0.209, 0.403]
Controlled motivation	Boys	3.06 (0.86)	3.62 (0.93)	0.006	[−956, −0.161]	3.48 (1.05)	3.44 (.87)	0.861	[−0.382, 0.456]
	Girls	2.83 (0.75)	3.17 (1.02)	0.113	[−757, 0.081]	3.26 (0.71)	3.34 (.89)	0.710	[−0.497, 0.340]
Amotivation	Boys	1.61 (0.70)	1.53 (0.60)	0.631	[−260, 0.427]	2.07 (1.20)	1.75 (1.06)	0.088	[−0.047, 0.677]
	Girls	1.45 (0.75)	1.34 (0.58)	0.544	[−251, 0.473]	1.66 (0.65)	1.66 (.70)	1.00	[−0.362, 0.362]
Perceptions of ability	Boys	3.68 (0.50)	4.36 (0.57)	<0.001	[−973, −0.401]	4.10 (0.61)	4.22 (.65)	0.437	[−0.420, 0.183]
	Girls	3.43 (0.65)	4.13 (0.64)	<0.001	[−998, −0.395]	3.65 (0.65)	3.85 (.60)	0.208	[−0.494, 0.109]
Intention to be physically active	Boys	3.84 (0.65)	4.62 (0.56)	<0.001	[−980, −0.427]	4.37 (0.57)	4.65 (.43)	0.046	[−0.558, −0.005]
	Girls	3.87 (0.69)	4.57 (0.53)	<0.001	[−558, −0.005]	4.02 (0.76)	4.34 (.56)	0.028	[−0.588, −0.034]

Note. *M* = mean; *SD* = standard deviation; CI = confidence interval.
